# Looking at the Pretty “Phase” of Membraneless Organelles: A View From *Drosophila* Glia

**DOI:** 10.3389/fcell.2022.801953

**Published:** 2022-02-07

**Authors:** Alexey L. Arkov

**Affiliations:** Department of Biological Sciences, Murray State University, Murray, KY, United States

**Keywords:** membraneless organelles, glia, germ granules, stress granules, Tudor domain, PIWI, neurodegenerative disease

## Abstract

Membraneless granules assemble in different cell types and cellular loci and are the focus of intense research due to their fundamental importance for cellular organization. These dynamic organelles are commonly assembled from RNA and protein components and exhibit soft matter characteristics of molecular condensates currently characterized with biophysical approaches and super-resolution microscopy imaging. In addition, research on the molecular mechanisms of the RNA–protein granules assembly provided insights into the formation of abnormal granules and molecular aggregates, which takes place during many neurodegenerative disorders including Parkinson’s diseases (PD), Alzheimer’s disease (AD), amyotrophic lateral sclerosis (ALS), and frontotemporal dementia (FTD). While these disorders are associated with formation of abnormal granules, membraneless organelles are normally assembled in neurons and contribute to translational control and affect stability of neuronal RNAs. More recently, a new subtype of membraneless granules was identified in *Drosophila* glia (glial granules). Interestingly, glial granules were found to contain proteins which are the principal components of the membraneless granules in germ cells (germ granules), indicating some similarity in the functional assembly of these structures in glia and germline. This mini review highlights recent research on glial granules in the context of other membraneless organelles, including their assembly mechanisms and potential functions in the nervous system.

## Introduction

Since description of characteristic membraneless granules in germ cells of over 80 animal species in eight phyla documented several decades ago (Eddy, 1975), it is now recognized that the assembly of large intracellular organelles, which lack the membrane, is one of the fundamental landmarks of cellular organization. In addition to formation of cell type-specific granules (such as germ granules in germ cells or neuronal granules in neurons), the membraneless structures are commonly assembled in nuclei and cytoplasm of most cells and include nucleoli, Cajal bodies, processing (P) bodies, and stress granules (Kiebler and Bassell, 2006; Anderson and Kedersha, 2008; Arkov and Ramos, 2010; Voronina et al., 2011; Gao and Arkov, 2013; Feric et al., 2016; Protter and Parker, 2016; Luo et al., 2018; Formicola et al., 2019; Marnik and Updike, 2019; Strom and Brangwynne, 2019; Trcek and Lehmann, 2019; Campos-Melo et al., 2021; Courchaine et al., 2021). In addition, multiple subcellular loci with crucial general functions such as silent chromatin (heterochromatin) and the RNA polymerase II-bound regions of transcriptionally active genes show properties of membraneless granules (Larson et al., 2017; Strom et al., 2017; Boehning et al., 2018; Lu et al., 2018). These soft matter properties are based on the granules’ assembly mechanisms which often can be described as protein/nucleic acid demixing (condensation) or phase separation from the rest of the cytosol (or nucleoplasm).

A recent study pointed to a number of common principles of the assembly of several membraneless ribonucleoprotein (RNP) granules. First, it was found that RNA components of the germ granules assemble in the large granule as homotypic clusters—large aggregates of the same RNA species (Little et al., 2015; Trcek et al., 2015; Trcek et al., 2020). In addition, some protein components of germ granules assemble into the granules as distinct clusters as well; however, the proteins show some overlap in the granules. Interestingly, the extent of the overlap for the same proteins and the distribution of the proteins within the granules can drastically change during development (Vo et al., 2019). Similar “docking” of two protein clusters, namely, coilin and survival of motor neuron (SMN) clusters, was recently described during the formation of Cajal body/gems granule in the nucleus (Courchaine et al., 2021). Also, a recent study provided a mechanistic view for the role of MEG-3 protein clusters, assembled at the interfaces of germ granules in *Caenorhabditis elegans* (P granules), in the regulation of granule condensates’ dynamics and stability (Folkmann et al., 2021). Interestingly, the function of these protein clusters is similar to the role of commonly used emulsion stabilizers (Pickering agents) adsorbed to interfaces of emulsion droplets.

Overall, for different membraneless granules, including germ granules, nucleoli, and Cajal bodies/gems, common structural features have emerged that show non-homogenous distribution of granule components within the granules, which can change during development and can be regulated by specific post-translational modifications of protein components. The functional significance and molecular mechanisms driving this non-random distribution of granule components is the subject of current research.

Many membraneless structures share landmark molecular characteristics, such as the presence of intrinsically disordered proteins (IDPs) or protein regions (IDPRs), which lack a defined folded structure, and low-complexity regions (LCRs), which may or may not have a distinct structure but show low diversity of their amino acids (Martin and Mittag, 2018). These protein regions are important components that can drive the assembly (condensation) of membraneless granules using their multivalent low-affinity associations with their interacting partners. Given the high occurrence of IDPs and IDPRs (about 51% of human proteins are either IDPs or contain IDPRs (Deiana et al., 2019)), it is surprising that different cell types use the same proteins to assemble membraneless granules. In particular, previous research works identified proteins that are found in both neuronal and germ granules, supporting the notion that neurons and germ cells utilize the same components to assemble the granules for specific similar functions in these cells (Kulkarni et al., 2020), and indicating that in addition to generally ubiquitous IDPRs, specifically structured protein domains may be selected to build the granules in different cells. Consistent with this, it was recently found that a structured Tudor (Tud) domain of SMN protein, and not its two IDPRs, is sufficient for formation of membraneless condensates (Courchaine et al., 2021).

In this mini review, the author highlights novel membraneless granules identified in glia of the adult *Drosophila* brain (referred to as “glial granules”) (Tindell et al., 2020), which were found to contain protein components of germ granules and discuss the potential significance of glial granules for brain functions in the context of other membraneless organelles.

## Glia and Glial Granules

### Glial Cells in *Drosophila*

Since one of the principal components of the *Drosophila* germ granules, Tud scaffold protein (Arkov et al., 2006; Gao et al., 2015; Vo et al., 2019), was found to be expressed not only in germline but also in the *Drosophila* head, distribution of Tud in the adult brain was recently examined in more detail (Tindell et al., 2020). These experiments led to the identification of Tud-containing large granules of about 0.5–2 μm in size in the cytoplasm of specific types of glial cells (but not in the neurons of the adult brain) (Tindell et al., 2020), Figure 1).

**FIGURE 1:**
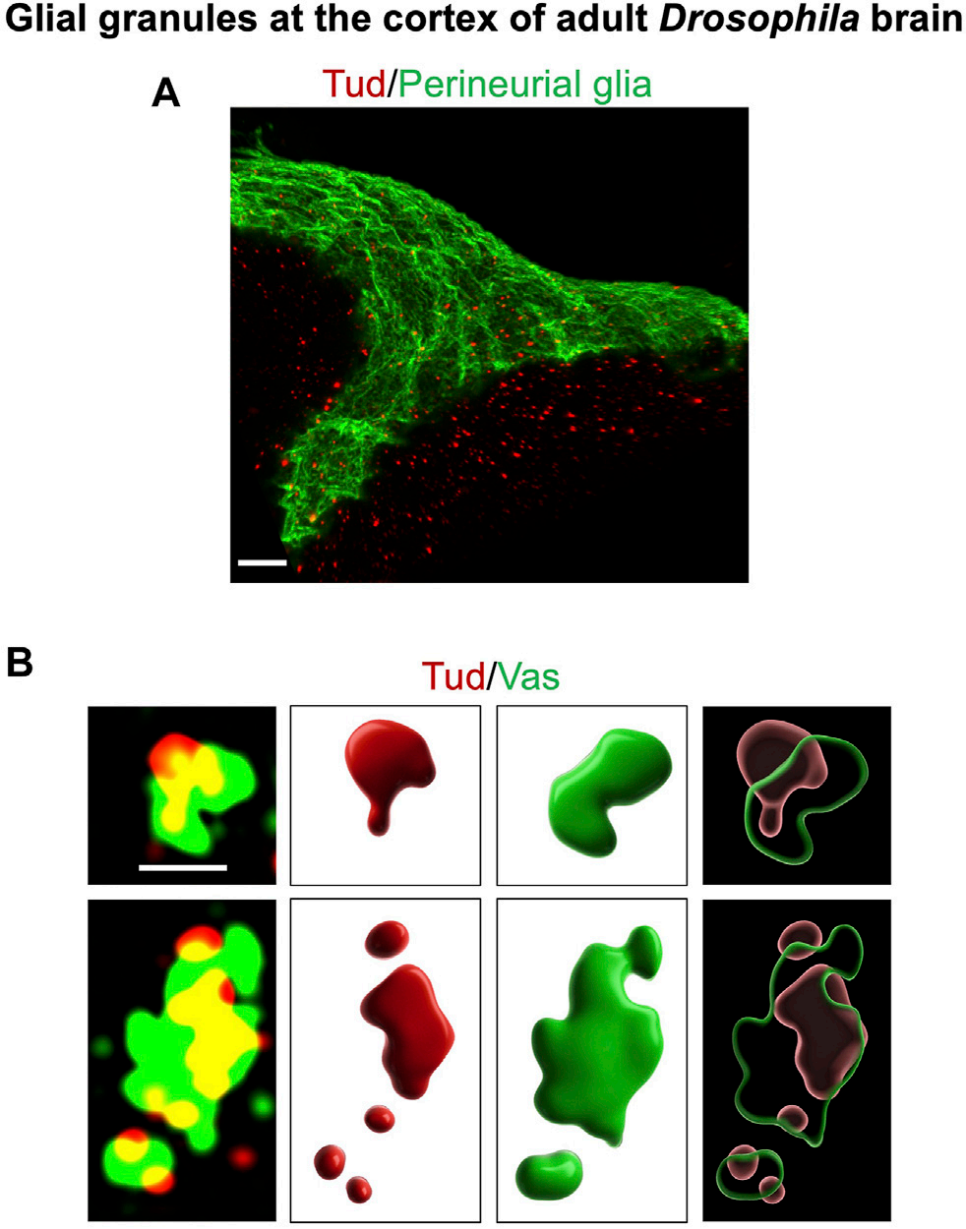
Glial granules in the *Drosophila* adult brain contain germline proteins, and they assemble from distinct protein clusters. This figure gives examples of glial granules recently published in Tindell et al. (2020). **(A)** A super-resolution microscopy image of a cortex region of the adult *Drosophila* brain shows multiple glial granules containing Tud protein (red channel) in surface glia (the outermost layer of surface glia, perineurial glia, is labeled with the membrane marker GFP-mCD8, green channel) and in the neighboring cortex glia. **(B)** Overlays of super-resolution optical sections of individual glial granules, which show Tud (red) and Vas (green) proteins (left panels), and corresponding 3D reconstructions of the individual protein clusters (middle panels) and composite granules (right panels) are shown. Scale bar in **(A)** is 7 μm. In **(B)**, scale bar, shown in the first optical section (top left), is the same for the other optical section and is 1 μm.

In the adult *Drosophila* brain, there are several types of glial cells, including surface glia, composed of perineurial and subperineurial glia, cortex glia, astrocyte-like glia, and ensheathing glia (Freeman, 2015; Kremer et al., 2017; Bittern et al., 2021). Interestingly, Tud-containing glial granules were observed in perineurial, subperineurial, and cortex glia, which are located at the surface and cortex of the brain. Perineurial and subperineurial glia form a barrier at the brain surface, and while the perineurial glia layer is porous and permeable, subperineurial glial cells, located below perineurial glia, are tightly connected with septate junctions, which prevent paracellular diffusion of the molecules, and thereby, surface glia is analogous to the blood–brain barrier in the mammalian brain. While both perineurial and subperineurial glia together form a continuous layer covering the entire central and peripheral nervous systems (Kremer et al., 2017), these glial cells employ different developmental strategies to form the layer. In particular, during development and expansion of the nervous system, perineurial glial cells constantly divide to produce a large number of relatively small narrow and elongated cells (Awasaki et al., 2008; Avet-Rochex et al., 2012). In contrast, subperineurial glial cells keep their small numbers during development but greatly enlarge their size by endoreplication (Unhavaithaya and Orr-Weaver, 2012).

As directly connected with hemolymph, perineurial glia is involved in transportation of nutrients from the hemolymph into the nervous system through its membrane transporters. In addition, perineureal glia plays an important role in ethanol tolerance using its A-kinase anchoring protein (Akap200)-dependent structural remodeling of actin cytoskeleton (Parkhurst et al., 2018). Interestingly, perineurial glia have circadian molecular clock (Zhang et al., 2018), and it is involved in the regulation of circadian locomotion behavior (Kozlov et al., 2020).

Subperineurial glia has been implicated in male courtship (Hoxha et al., 2013) and sleep behavior (Artiushin et al., 2018). In addition, subperineurial glia’s efflux transporters are regulated by the circadian clock in perineurial glia, and they are responsible for pumping xenobiotics out into hemolymph most actively during daytime (Zhang et al., 2018).

**FIGURE 2:**
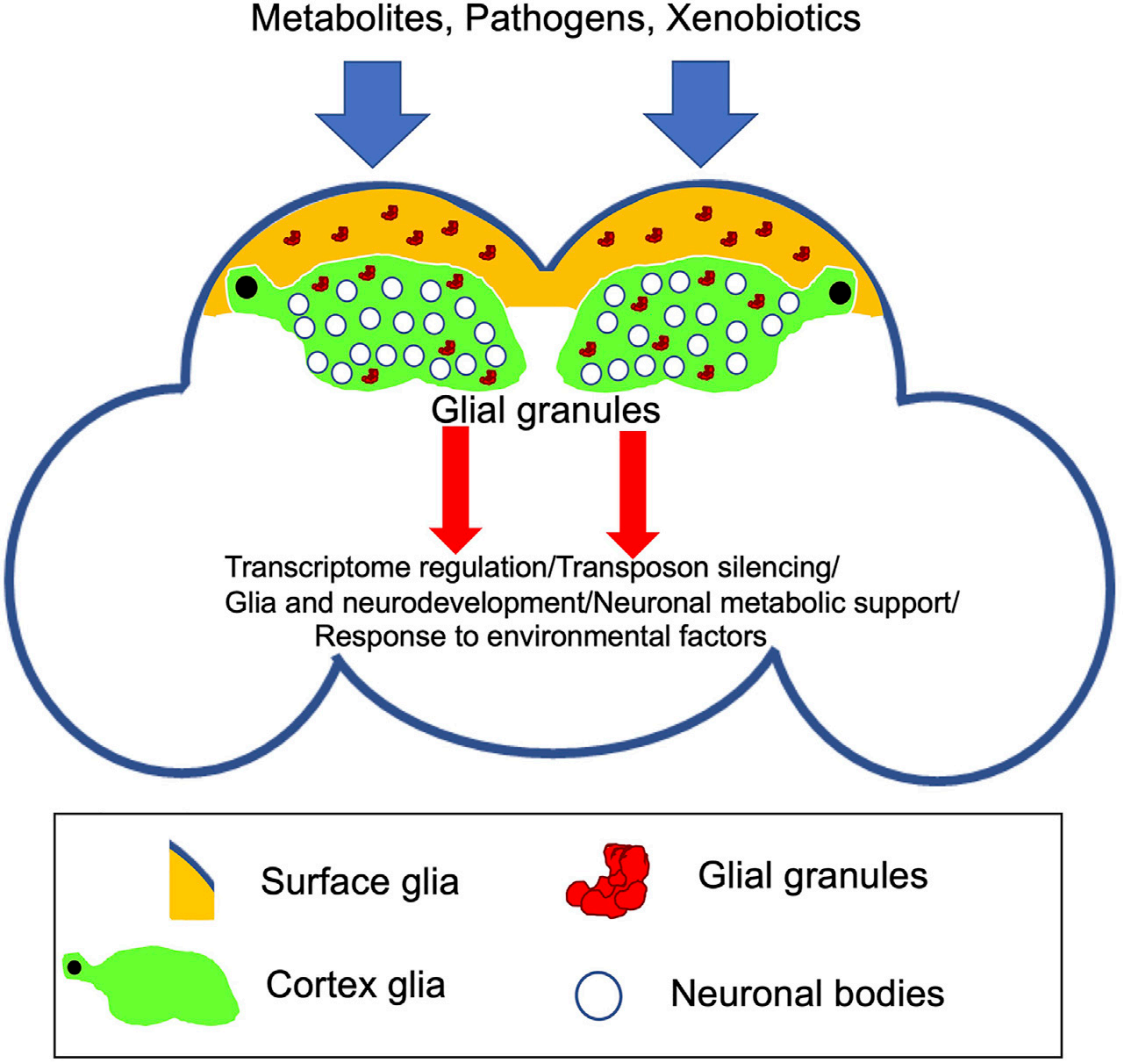
Schematic of the adult *Drosophila* brain indicating novel glial granules assembled in surface and cortex glia. Recent evidence points to the function of these granules in transcriptome regulation and silencing of transposable elements in the *Drosophila* brain (Tindell et al., 2020). Future research will test whether glial granules are involved in specific aspects of brain development and function including neuronal survival and metabolic support, behavior, and response to external/environmental factors.

Cortex glial cells encapsulate virtually all neuronal cell bodies in the fly brain (Figure 2) and are functionally equivalent to mammalian protoplasmic astrocytes (Pereanu et al., 2007; Awasaki et al., 2008; Freeman, 2015). Cortex glia are involved in several essential physiological and behavioral aspects such as metabolic neuronal support, control of neuronal excitability, sleep, locomotion, ethanol response, and removal of dead neurons using phagocytosis (Coutinho-Budd et al., 2017; Farca Luna et al., 2017; Delgado et al., 2018; Lee et al., 2019; Mclaughlin et al., 2019; Nakano et al., 2019; Weiss et al., 2019). In addition, defects in the membrane of the cortex glia cause light-induced seizures (photosensitive epilepsy) (Kunduri et al., 2018). Interestingly, a recent study demonstrated that fragile X mental retardation protein (FMRP), which is one of the crucial components of neuronal granules, has an additional role in glia-mediated phagocytosis that is responsible for neuronal clearance after injury and during neurodevelopment (O’Connor et al., 2017). Importantly, during development, neuronal FMRP signals to cortex and ensheathing glia to activate glial phagocytosis to carry out removal of transient neurons (Vita et al., 2021).

### Germline Components of Glial Granules

#### Tudor and Tudor Domains

Tud protein is one of the principal components of germ granules and plays a role of molecular scaffold due to its 11 protein–protein interaction modules (Tud domains) (Arkov et al., 2006; Liu et al., 2010; Gao et al., 2015; Zheng et al., 2016). The Tud domain is a 50–55 amino acid β-barrel structure, which forms an interaction cavity referred to as “aromatic cage” since it is lined with aromatic amino acids. Aromatic cage of the Tud domain interacts with methylated amino acids of Tud partner proteins such as methylated arginines and lysines. In germ cells, Tud is required for the assembly of germ granules at the posterior pole of the egg (polar granules), and it is essential for formation of primordial germ cells during early embryogenesis (Boswell and Mahowald, 1985; Thomson and Lasko, 2004; Arkov et al., 2006). Tud domain-containing proteins are molecular landmarks of germline in many animals and important contributors to the mechanisms responsible for silencing of transposable elements (Arkov and Ramos, 2010). Similarly, glial granule Tud protein is required for silencing of transposable elements in the *Drosophila* adult brain (Tindell et al., 2020).

Interestingly, neuronal granule FMRP contains two tandem domains of Tud family (Ramos et al., 2006; Myrick et al., 2015). In *Drosophila* germ cells, FMRP homolog was also found to associate with Piwi family proteins, Piwi and Aubergine (Aub), and contribute to silencing of transposable elements (Megosh et al., 2006; Bozzetti et al., 2015).

#### Piwi Family Proteins

*Drosophila* genome encodes three Piwi family proteins (Piwi, Ago3, and Aub), which belong to a distinct clade of a larger group of argonaute (Ago) proteins (Ku and Lin, 2014; Iwasaki et al., 2015; Arkov, 2018). Similar to other Argonautes, which associate with small guide RNAs, Piwi proteins associate with 24–31 nucleotides long Piwi-interacting RNAs (piRNAs). In germline, piRNAs guide Piwi proteins to the transposon RNA targets, and thereby Piwi proteins silence these targets by either direct endonucleolytic cleavage (Nishida et al., 2015) or by repressing transcription of transposon genes by the assembly of heterochromatin at transposon genomic loci (Le Thomas et al., 2013).

In different animals, Tud domain-containing proteins interact with symmetrically dimethylated arginines (sDMAs) of Piwi proteins in germ granules (Kirino et al., 2009; Vagin et al., 2009). In particular, in *Drosophila* germline, methylated Aub associates with Tud domain proteins Tud and Krimper (Nishida et al., 2009; Kirino et al., 2010; Huang et al., 2021; Vrettos et al., 2021). In glial granules, both Piwi and Ago3 were identified; however, Aub protein was not detected in these granules. In addition to Ago3 assembly in glial granules, Ago3 was also expressed in a distinct population of neurons (Tindell et al., 2020). Consistent with these data, previous study showed expression of Aub and Ago3 in neurons of mushroom body, which is crucial for olfactory memory, and provided genetic evidence that, similar to the role of Aub and Ago3 in germ cells, these proteins repress transposable elements in the adult brain (Perrat et al., 2013). Interestingly, Aub principal interacting partner in germ granules, Tud protein, is not detected in neurons of the adult brain, and whether Aub is assembled in neuronal granules in mushroom body and associates with other Tud domain-containing proteins awaits further investigation.

#### ATP-Dependent RNA Helicase Vasa

Vasa (Vas) is a DEAD-box RNA helicase, which is required for development and specification of germline in different organisms (Raz, 2000) (see Figure 1B, which shows Vas and Tud in glial granules (Tindell et al., 2020)). Similar to Tud domain and Piwi proteins, Vas is essential for silencing of transposable elements in germline and is one of the principal components of germ granules (Dehghani and Lasko, 2017). Generally, in the granules, Vas may use its RNA helicase activity to remodel RNA in ribonucleoprotein complexes and to function as an RNA chaperone and regulator of RNA stability and translation (Sengoku et al., 2006; Arkov and Ramos, 2010). In particular, it was shown that Vas helps to dissociate target RNA fragments from Aub homolog from *Bombyx mori* (silkworm) (Siwi) after Siwi’s cleavage of its target RNA, thereby increasing the efficiency of transposon silencing mechanisms (Nishida et al., 2015; Arkov, 2018).

#### Polar Granule Component

The polar granule component (pgc) encodes a 71 amino acid protein, and in germ cells it localizes to the nuclei where it represses elongation of transcription by preventing the action of positive transcription elongation factor (P-TEFb). P-TEFb is composed of cyclin-dependent kinase 9 (Cdk9) and cyclin T (CycT), and Pgc protein binds to Cdk9 subunit of the P-TEFb complex, which interferes with association of P-TEFb with transcriptionally active regions (Hanyu-Nakamura et al., 2008). Recently, Pgc was shown to play a role in repressing production of miRNA precursors in germ cells, thereby enabling expression of germline mRNAs, which are targeted by miRNAs, thus preventing germ cell death during embryogenesis (Hanyu-Nakamura et al., 2019). While all the reported functions of Pgc in germ cells are associated with its role in transcription, identification of Pgc protein in cytoplasmic glial granules may point to a novel role of this protein in glia. Alternatively, Pgc protein may be stored in glial granules and, upon a signaling event in glia, Pgc could be released from the granules and translocated to the nucleus for transcriptional regulation.

## Functional Aspects of the Membraneless Organelles

Different membraneless granules have been implicated in a variety of functions, including regulation of translation and RNA storage/localization (P-bodies, stress granules, germ granules, and neuronal granules) (Arkov and Ramos, 2010; Voronina et al., 2011; Gao and Arkov, 2013; Luo et al., 2018; Formicola et al., 2019; Marnik and Updike, 2019; Trcek and Lehmann, 2019; Campos-Melo et al., 2021), compartmentalization of distinct steps of ribosome assembly in nucleoli (Feric et al., 2016), and bringing together different piRNA pathway components to enable efficient silencing of transposable elements in germ granules (Arkov, 2018).

In particular, in the nervous system, neuronal or transport membraneless granules are assembled to control translation of neuronal mRNAs. One of the principal components of neuronal granules, FMRP, is a well characterized RNA-binding protein and translational repressor (Tsang et al., 2019; Lai et al., 2020). Upon the assembly of the granules, translation of multiple neuronal mRNAs is repressed during transport of these mRNAs from neuronal cell body to the synaptic regions. When granules arrive in the synaptic region, they release or unmask these mRNAs for local translation. Interestingly, there is evidence that this mRNA unmasking and translational activation during neuronal stimulation is promoted by disassembly of the neuronal granules which is induced by dephosphorylation and methylation of FMRP, decreasing its property to form RNA–protein condensates by phase separation (Tsang et al., 2019).

Similar to neuronal granules, stress granules repress translation of their RNA components upon various stress conditions and can transfer the RNAs to P-bodies for degradation of these RNAs (Anderson and Kedersha, 2008). However, recently it was also shown that some RNAs in stress granules are translationally active (Mateju et al., 2020). Importantly, dysregulation of RNA–protein granules, in particular, stress granules, has been strongly implicated in development of several neurodegenerative diseases, such as Alzheimer’s, Huntington’s, ALS, and FTD, for which there is currently no cure (Ash et al., 2014; Mcaleese et al., 2017; St-Amour et al., 2018; Coudert et al., 2019; Ryan and Fawzi, 2019; Wolozin and Ivanov, 2019; Dudman and Qi, 2020). Furthermore, mutations in genes encoding stress granule RNA-binding proteins including TAR-DNA binding protein (TDP-43), fused in sarcoma/translated in liposarcoma (FUS/TLS), heterogeneous nuclear ribonucleoprotein A1 (hnRNPA1), and T cell-restricted intracellular antigen-1 (TIA-1) have been found in ALS/FTD patients pointing to direct link between abnormal regulation of stress granule formation in these neurodegenerative disorders (Benajiba et al., 2009; Kwiatkowski et al., 2009; Pesiridis et al., 2009; Gendron et al., 2013; Kim et al., 2013; Mackenzie et al., 2017). Importantly, many of these mutations in stress granule proteins are found in their LCRs, which result in the granule’s decreased dynamics and increased liquid–liquid phase separation, and ultimately, it leads to irreversibly insoluble pathological granules (Baradaran-Heravi et al., 2020).

While glial granules have been described very recently, it is possible that they are involved in post-transcriptional gene regulation in glia, including translational repression, RNA degradation, and recruitment of other RNAs for storage. It is intriguing that Tud-containing glial granules have been detected only in surface and cortex glia, which may indicate that the granules have roles important for the specific functions of these types of glia located at or close to the surface of the brain. For example, these functions may be related to the brain response to or protection from the environmental factors or pathogens present in hemolymph and metabolic support of neurons (Figure 2). In addition, to prevent a pathological cell transformation**,** co-expression of germline genes in somatic cells may need to be restricted to and tightly controlled in a limited group of cells when germ-like granules are needed for function since ectopic expression of several germline components of glial granules including Piwi and Vas has been observed in *Drosophila* larval brain tumors in *lethal (3) malignant brain tumor* (*l(3)mbt*) mutants (Janic et al., 2010). Importantly, ectopic expression of these genes contributes to tumor development in the *l(3)mbt* mutants indicating that the germline genes may act to drive tumorigenesis in somatic cells.

Transcriptomic data showed that glial granule component Tud is required for silencing of transposable elements in the brain (Tindell et al., 2020). Consistent with these data, Piwi proteins, Piwi, and Ago3, which are the principal factors of the transposon silencing mechanisms in the germ granules, are bona fide components of glial granules. In addition, non-transposon RNAs are likely to be regulated in glial granules also, which is consistent with the finding that *tud* mutant showed significant misregulation of multiple non-transposon RNAs in the *Drosophila* brain (Figure 2).

## Future Perspectives

In conclusion, while it is intriguing that several proteins are shared by glial and germ granules, these granules are likely to include specific components needed for their functions in glia and germline, respectively. Therefore, future research will comprehensively analyze the glial granule composition and test functional roles of the granule components in the brain. In particular, future studies on glial granules should determine whether specific neuron/glia or glia/hemolymph signaling affects the assembly and potential roles of these novel granules in post-transcriptional gene regulation, response to environmental factors, and behavior (Figure 2). Furthermore, potential functional links between glial granules and other membraneless organelles, such as stress granules, should be explored. In addition, given the importance of glia in virtually every aspect of neuronal activity, it will be important to determine whether glial granules show age-related changes in dynamics and composition, which may be linked to neurodegeneration. Overall, future research works on glial granules may provide important insights into biogenesis and formation of pathological granules in the nervous system and suggest ways to reverse their assembly.
